# Microglia and cognitive impairment in schizophrenia: translating scientific progress into novel therapeutic interventions

**DOI:** 10.1038/s41537-023-00370-z

**Published:** 2023-07-10

**Authors:** Chuanjun Zhuo, Hongjun Tian, Xueqin Song, Deguo Jiang, Guangdong Chen, Ziyao Cai, Jing Ping, Langlang Cheng, Chunhua Zhou, Chunmian Chen

**Affiliations:** 1 Key Laboratory of Sensory Information Processing Abnormalities in Schizophrenia (SIPAS-Lab), Nankai University Affiliated Tianjin Fourth Center Hospital, Tianjin Medical University Affiliated Tianjin Fourth Center Hospital, Tianjin Fourth Center Hospital, Tianjin, China; 2 Laboratory of Psychiatric-Neuroimaging-Genetic and Co-morbidity (PNGC-Lab), Nankai University Affiliated Tianjin Anding Hospital, Tianjin Mental Health Center of Tianjin Medical University, Tianjin Anding Hospital, 300222 Tianjin, China; 3 Department of Psychiatry, the First Affiliated Hospital of Zhengzhou University, Zhengzhou, China; 4 Department of Psychiatry, Wenzhou Seventh peoples Hospital, Wenzhou, China; 5 Henan International Joint Laboratory of Biological Psychiatry, Zhengzhou, China; 6 Henan Psychiatric Transformation Research Key Laboratory, Zhengzhou University, Zhengzhou, China; 7 Department of Pharmacology, The First Hospital of Hebei Medical University, Shijiazhuang, China

**Keywords:** Schizophrenia, Neuroscience

## Abstract

Cognitive impairment is a core clinical feature of schizophrenia, exerting profound adverse effects on social functioning and quality of life in a large proportion of patients with schizophrenia. However, the mechanisms underlying the pathogenesis of schizophrenia-related cognitive impairment are not well understood. Microglia, the primary resident macrophages in the brain, have been shown to play important roles in psychiatric disorders, including schizophrenia. Increasing evidence has revealed excessive microglial activation in cognitive deficits related to a broad range of diseases and medical conditions. Relative to that about age-related cognitive deficits, current knowledge about the roles of microglia in cognitive impairment in neuropsychiatric disorders, such as schizophrenia, is limited, and such research is in its infancy. Thus, we conducted this review of the scientific literature with a focus on the role of microglia in schizophrenia-associated cognitive impairment, aiming to gain insight into the roles of microglial activation in the onset and progression of such impairment and to consider how scientific advances could be translated to preventive and therapeutic interventions. Research has demonstrated that microglia, especially those in the gray matter of the brain, are activated in schizophrenia. Upon activation, microglia release key proinflammatory cytokines and free radicals, which are well-recognized neurotoxic factors contributing to cognitive decline. Thus, we propose that the inhibition of microglial activation holds potential for the prevention and treatment of cognitive deficits in patients with schizophrenia. This review identifies potential targets for the development of new treatment strategies and eventually the improvement of care for these patients. It might also help psychologists and clinical investigators in planning future research.

## Introduction

Schizophrenia, a severe psychiatric disorder, remains an etiological and therapeutic challenge worldwide. Patients with schizophrenia present emotional and behavioral dysfunction, and usually some degree of cognitive deficit^1^. Cognitive impairment is well recognized as a core clinical feature of this disorder that has profound adverse impacts on the social functioning and quality of life of a majority of patients with schizophrenia^1,2^. Its trajectory has been shown to be distinct from those of the other clinical features of schizophrenia; it may be pre-existing and worsen abruptly within months before the appearance of psychotic symptoms^1,3^. No drug is currently available for the treatment of cognitive impairment in patients with schizophrenia. Although the pathological mechanisms underlying this impairment remain to be elucidated, substantial progress has been made in our understanding of causal factors and identification of targets for the development of novel therapeutic or even preventive interventions.

Microglia, together with astroglia, satellite glia, and oligodendroglia, are non-neuronal glial cells in the brain. Scientists are gaining increasing insight into the roles of microglia; they are known to have an immunological heritage and to act as primary resident macrophages, maintaining brain function and playing important roles in complex psychiatric disorders, including schizophrenia^4,5^. Microglia have immune and multiple non-immune functions essential for brain development and homeostasis. For instance, they directly mediate normal synaptic reorganization (pruning) in the developing brain, thereby playing a critical role in the modulation of synapse plasticity, defined as the ability of neurons to change the strength or efficacy of communication via synaptic transmission^5,6^. Using an in-vitro model of microglia-mediated synaptic pruning established with patient-derived neural cultures and isolated synaptosomes, Sellgren et al.^6^ obtained results suggesting that schizophrenia-related risk variants in the *C4* locus lead to *C4A*-mediated excessive complement deposition, which may contribute, at least in part, to increased synaptic pruning mediated by activated microglia in schizophrenia. The exact mechanisms by which microglial activation is related to synaptic pruning and cognitive deficits in schizophrenia remain unclear, and further in-depth research is needed to provide fundamental evidence for the associations being suggested. Synapse plasticity is believed to be among the most fundamental abilities of the brain and a cellular basis for memory and learning^5,6^. This predominant type of non-neuronal glial cell is thus hypothesized to be linked to the pathogenesis of schizophrenia and the presence of cognitive impairment before or at the time of the first schizophrenic episode^4,7^. Findings from several studies support this hypothesis. For instance, postmortem histological examination and brain imaging revealed aberrant microglial activated in the brains of patients with schizophrenia, especially in the hippocampus, which is known to be associated with memory^8–11^. In addition, levels of key microglia-produced proinflammatory cytokines, including interleukin (IL)−6, tumor necrosis factor-alpha (TNF-α), and IL-1β, were elevated and these higher levels were correlated with poorer cognitive performance in patients with schizophrenia^12,13^. Rapid progress is being made in understanding the roles of microglia in this context, including abnormal activation, and the identification of microglia-expressed genetic variants and gray matter (GM)-mediated associations. Most recently, Corley and colleagues^14^ identified microglial-expressed risk-related genetic variants affecting the general cognitive ability, episodic memory, and working memory of patients with schizophrenia. In addition, higher microglial polygenic scores (PGSs), used to measure schizophrenia risk–associated microglial gene expression, were associated with greater cognitive impairment and lower gray matter volumes (GMVs) in patients with schizophrenia and in a large independent sample of UK Biobank participants (*n* = 134,827)^14^. These findings provide direct evidence of the contribution of genes expressed in the microglia to cognitive impairment in schizophrenia^14^. Research on correlations of microglial gene expression, molecular pathways, and brain structural and functional imaging findings with cognitive impairment is still in its infancy, and further in-depth investigation of the underlying mechanisms may aid the development of novel treatments for cognitive impairment in patients with schizophrenia.

We conducted this review to highlight recent findings regarding the roles of resident immune cells in the brain and microglia in cognitive deficits relevant to schizophrenia, and to discuss the aberrant activation of microglia even in the premorbid phase of schizophrenia. The gaining of insight into the pathological mechanisms underlying cognitive impairment in schizophrenia is a high research priority in the field, and discoveries have the potential to be translated into novel preventive and therapeutic measures. As the translation of such findings into therapeutics may involve the overcoming of certain challenges and the resolution of scientific issues, we also discuss future research directions to accelerate therapeutic development. Thus, this review may help physicians and clinical investigators in the field plan future research.

### Cognitive impairment in schizophrenia

The German psychiatrist Emil Kraepelin (1856–1926) initially described cognitive impairment in patients with schizophrenia as dementia praecox^15^. Although cognitive impairment has not been included as a diagnostic criterion for schizophrenia in major guidelines (e.g., the International Classification of Diseases and Diagnostic and Statistical Manual of Mental Disorders), it is a well-documented core symptom of the disorder, as demonstrated by multiple lines of evidence, including but not limited to (1) the detection of cognitive decline relative to the premorbid status in up to 98% of patients with schizophrenia^15,16^, (2) the detection of cognitive deficits of varying severity in multiple domains in patients with schizophrenia^2,15^, and (3) the presence of cognitive impairment in the premorbid stage and at the onset of psychosis before treatment with antipsychotic medications in these patients^17^. Cognitive impairment has been found to progress in the first 3 years after the initiation of treatment with currently available antipsychotic agents and after remission in patients with schizophrenia^18–20^, suggesting that schizophrenia-related cognitive impairment has a course distinct from that of psychotic symptoms in these patients. The magnitude of cognitive impairment in schizophrenia varies across the premorbid phase, the time of the first psychotic episode, during treatment (or lack thereof) with antipsychotic medication, and after remission.

Neurocognitive dysfunction is a common symptom of schizophrenia, bipolar disorder (BD), and major depressive disorder (MDD), and comparisons of its profile and severity in these conditions have been extensive and have yielded somewhat mixed results. Cognitive deficits have been consistently found to be more severe and prevalent (affecting >80% of individuals) in patients with schizophrenia than in those with BD and MDD^15,21–27^. Furthermore, cognitive impairment in patients with schizophrenia usually precedes the first psychotic episode and subsequent diagnosis of the disease^28,29^. In contrast, better and worse premorbid cognitive performance has been associated with an increased risk of BD^30–32^. The reason underlying this difference is poorly understood. In a recent genome-wide analysis of a large sample of patients with schizophrenia (*n* = 82,315), those with BD (*n* = 51,710), and control individuals (*n* = 269,867), Smeland et al.^22^ revealed substantial overlap and distinctiveness of genetic factors related to cognitive performance in the former two groups. The majority (81%) of schizophrenia risk alleles correlated with lesser cognitive performance, whereas 75% of BD risk alleles were related to better cognitive performance^22^. This important study demonstrated the genetic basis, at least in part, of the shared but distinct cognitive impairment in patients with schizophrenia and those with BD^22^.

Schizophrenia-related cognitive impairment commonly affects seven domains of cognition: processing speed, attention and concentration, working memory, verbal learning and memory, visual learning and memory, reasoning and problem solving, and social cognition^15,33–35^. In a recent comprehensive examination of the findings of previous meta-analyses and systematic reviews, Gebreegziabhere and colleagues^15^ found that processing speed, working memory, and verbal learning and memory were more impaired than other cognitive domains, and that greater cognitive impairment was associated with poorer insight and functionality, in patients with schizophrenia. At present, the underlying mechanisms remain unclear. Functional imaging and neuropsychological findings have linked the loss of GMV to cognitive impairment in patients with schizophrenia^19,20^. In contrast to the age-related cognitive impairment occurring in Alzheimer’s disease, the impairment occurring in schizophrenia is often characterized by a greater loss of GMV than of white matter volume (WMV)^19,20^. The differences in the domains of cognition affected, the magnitude of cognitive impairment, and brain structural and functional changes may mean that the pathological mechanisms implicated in schizophrenia-related cognitive impairment differ from those implicated in other forms of such impairment.

Hypothetical explanations for schizophrenia-associated cognitive impairment involve the aberrant activation of microglial cells^4,5^. In view of the emerging link of microglial activation to cognitive impairment in schizophrenia, we next focus on current insights into the roles of microglia and potential underlying mechanisms.

### Progress in understanding the roles of microglia in cognitive impairment in schizophrenia

The past two decades have witnessed a revolutionary discovery and subsequent advances in our understanding of microglia, including the association of pro-inflammatory polarization and excessive microglial activation with synaptic pruning in juveniles. Synaptic pruning is the process of eliminating extra neurons and synaptic connections and is essential for the maintenance of normal brain development, particularly in the early postnatal period and adolescence in animals and humans. Extensive synapse elimination in the cerebral cortex usually occurs in late adolescence^36^. Exaggerated synaptic pruning by microglia has been proposed to be a key contributing factor and potentially involved in the pathogenesis of schizophrenia^6,37,38^. The roles of microglia in the development and worsening of cognitive impairment and other symptoms of schizophrenia are increasingly being uncovered^4,5^. Substantial progress has been made in determining how microglial cells are activated, how homeostasis is modulated, and how microglial activation influences synaptic pruning, synapse plasticity, and the phagocytosis of apoptotic cells in the brain^4,5,7^.

#### Microglial activation in schizophrenia

Bloomeld and colleagues^39^ showed that the binding ratio of the second-generation translocator protein (TSPO) radioligand [^11 ^C] peripheral benzodiazepine receptor 28 (PBR28), a noninvasive measure reflecting the number of activated microglia on brain positron emission tomography (PET), was significantly greater in the GM of patients with schizophrenia and individuals at ultra-high risk of psychosis (i.e., unmedicated individuals with sub-clinical symptoms) than in matched healthy controls. Moreover, this ratio correlated positively with the severity of symptoms in patients with schizophrenia^39^. Similarly, another PET study showed increased TSPO ([^11 ^C]PBR28) binding in patients with schizophrenia, correlated negatively with the GM volume^40^. These studies provided evidence that microglial activity is increased in individuals with schizophrenia and those at ultra-high risk of psychosis, and that microglia-mediated neuroinflammation is associated with the risk of psychosis, and possibly schizophrenia, development in later life^39,40^. However, PET studies conducted with different TSPO radioligands have yielded different results for patients with schizophrenia and individuals at high risk of psychosis^40–42^. For instance, Hafizi et al. performed PET imaging study using TSPO radioligand found no difference in the microglial activity of untreated patients experiencing their first psychotic episodes and healthy controls^41^ in a study in which [^18 ^F]FEPPA was used as the radioligand. In addition to the radioligand difference, such differences may be related to the examination of small samples and the inclusion of patients taking atypical antipsychotics or with schizophrenia of different stages.

More recently, Gober et al.^43^ examined microglial activity and morphological alterations postmortem in the brains of 16 patients with schizophrenia and 13 controls through density quantification and three-dimensional reconstruction for multiple regions of interest (temporal, frontal, and subcortical WM regions; cingulate cortical GM; and anterior corpus callosum). They revealed increased microglial density in the cortical GM, temporal, and frontal regions in patients with schizophrenia^43^. The main morphological alterations detected in these patients were region-dependent increases in microglial soma size^43^. In another postmortem study, De Picker et al. reported that the expression of microglial Fc gamma (Fcγ) receptors, including CD64 (Fcγ RI) and CD64/human leukocyte antigen-DR isotype, was increased in the dorsal prefrontal cortex of patients with schizophrenia^44^. Considering this finding, together with earlier postmortem brain imaging and histological findings from various research groups^8,10,11,13^, which may be affected by limitations and are inconsistent, we are inclined to infer that microglial activation is abnormally excessive in a subset of patients with schizophrenia.

#### Potential role of microglial activation in schizophrenia-related cognitive deficits

Microglial activation is a hallmark of neuroinflammation that occurs predominantly in brain regions related closely to memory, such as the hippocampus^8–11^. Activated microglia produce and secrete key proinflammatory cytokines, including IL-6, IL-1β, and TNF-α^12,13^. They also release free radicals, such as reactive oxygen and nitrogen species, which are well-recognized toxic factors contributing to cognitive degeneration. Based on this knowledge, a better understanding of the pathophysiological mechanisms of microglial activation and related neuroinflammation in schizophrenia is anticipated to lead to the identification of microglia-targeting novel therapeutics. In view of the high degree of schizophrenia heritability, which accounts for approximately 60–80% of cases of this disorder, genome-wide association studies have been conducted. The largest such study to date resulted in the identification of 108 schizophrenia-associated genetic loci, including a major histocompatibility complex locus on chromosome 6, related most significantly to schizophrenia^45^. Most recently, Corley and colleagues^14^ identified microglia-expressed risk-related genetic variants affecting three cognitive domains (general cognitive ability, episodic memory, and working memory) in patients with schizophrenia. They reported that higher microglial PGSs, used as a measure of schizophrenia risk–associated microglial gene expression, were associated with worse cognitive impairment and lesser GMVs in patients with schizophrenia^14^. These findings provide direct evidence that microglia-expressed genes contribute to cognitive impairment in schizophrenia^14^.

Microglial activation has been linked clearly to schizophrenia-related cognitive dysfunction, and rapid progress has been made in the identification of microglia-expressed genetic variants and the GM-mediated association between microglial activation and cognitive impairment in schizophrenia. In contrast to age-related cognitive impairments, such as Alzheimer’s disease, in which roughly three times more WMV than GMV is lost, the loss of GMV is the predominant brain structural change in schizophrenia. Research on correlations of microglia-expressed genes, molecular pathways, and brain structural and functional imaging alterations with cognitive impairment is in its infancy, and such investigation may facilitate the elucidation of pathological mechanisms and thereby aid the development of novel schizophrenia treatments.

#### Roles of microglial gene expression in microglial activation–mediated cognitive deficits, especially memory impairment, in schizophrenia

Corley et al.^14^ were the first to profile all microglia-expressed genes and variations and to assess their associations with cognitive function in patients with schizophrenia relative to that in matched healthy controls. They identified complement-related genes associated with cognition in schizophrenia^14^, consistent with previous findings^46–50^. Earlier genetic association studies revealed that complement component 4^46,47^, the complement-regulating genes CUB and Sushi Multiple Domains 1 and 2^49^, and complement factor H, a regulator of the complement activation gene cluster^50^, were associated with cognitive function, especially memory, in patients with schizophrenia. Excessive microglial activation, but not tau pathology, has also been observed to adversely affect memory function in patients with Alzheimer’s disease, with an independent association between aberrant microglial activation and memory impairment^51^; this excessive activation has been demonstrated to co-occur with brain structural changes and memory deficits^52^. Corley et al.^14^ calculated PGSs, estimates of the genetic risk of a disease or trait, for the schizophrenia-related microglial gene set (SZ-PGSs). These scores predicted variation in measures of cognition (e.g., full-scale IQ, performance IQ, memory) relative to healthy controls across three domains: general cognitive ability, episodic memory, and working memory^14^. Furthermore, higher microglial SZ-PGSs correlated with worse cognitive performance and the strongest association was observed for memory^14^. These results are in line with the previous finding that memory function impairment is the most consistently observed schizophrenia-related cognitive impairment^53^. These studies have provided scientific evidence supporting the association between greater microglial polygenic risk and worse cognitive performance, especially memory function, in patients with schizophrenia. Unlike microglial SZ-PGSs, astroglial SZ-PGSs were not associated with cognitive performance in patients with schizophrenia^14^, suggesting that this association is specific to microglia. Few studies to date have involved the examination of the relationship between brain structure and microglial abnormalities; Corley et al.’s^14^ findings suggest that microglial overactivation is linked to brain structural (i.e., GMV) and functional (i.e., cognitive processing) changes, and that the GMV mediates the association between microglial SZ-PGSs and cognitive performance. Numerous neuroimaging findings support the notion that abnormal GMV alterations in the whole brain form the neural basis of cognitive impairment^1,53^. We found that the baseline global GMV in drug-naïve patients experiencing their first episodes of schizophrenia was associated with the severity of cognitive deterioration after 2 years of treatment with antipsychotic drugs^18^. Collectively, these findings suggest that the GMV mediates the association between the microglial SZ-PGS and cognitive impairment in schizophrenia.

#### Antipsychotic drugs may affect microglial activation in patients with schizophrenia

At present, evidence from clinical studies is insufficient to determine the effect of antipsychotic drugs on the cognitive ability of patients with schizophrenia. In a recent study, we demonstrated that global GMV alterations at baseline in drug-naïve patients experiencing their first episodes of schizophrenia were associated with the severity of cognitive alterations [evaluated using the Measurement and Treatment Research to Improve Cognition in Schizophrenia (MATRICS) consensus cognitive battery and global deficit score] after 2 years of antipsychotic treatment, and that cognitive impairment had worsened from baseline in these patients after antipsychotic therapy of adequate dosage and duration^18^.

Clinical data have not clarified the effect of antipsychotic agents on microglial cells in the brain.^54^ In-vivo neuroimaging and postmortem studies of patients with schizophrenia, as well as animal models of schizophrenia, have suggested that antipsychotic agent exposure affects the microglia in the brain^54^. For instance, Cotel et al. reported that chronic exposure to clinically comparable antipsychotic (i.e., haloperidol and olanzapine) doses may alter the density and morphology of microglia in the rat brain^55^. The molecular mechanisms underlying these effects, and the clinical relevance of the observed beneficial and side effects of antipsychotic drug regimens, remain unclear; further investigation is warranted^54,55^.

**Fig. 1:**
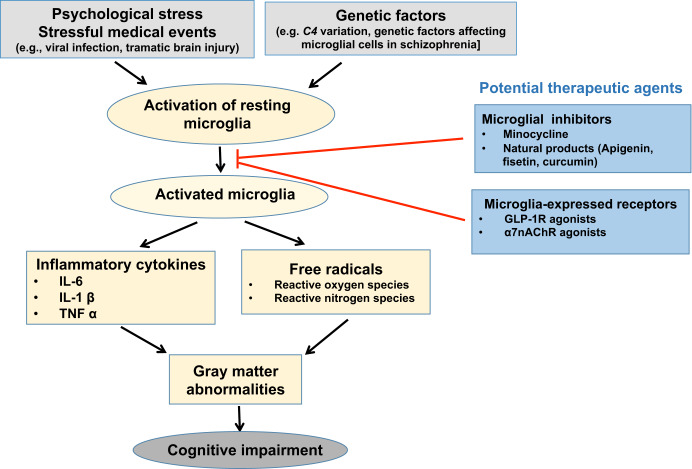
Schematic of the hypothetical model of microglial activation and cognitive impairment in schizophrenia, and potential therapeutic agents. Microglial activation in the brain is induced by various stressful events, mainly psychological stress and events such as viral infection or traumatic brain injury, which usually occur prior to the onset of psychotic symptoms in schizophrenia. In addition, genetic factors [i.e., complement component (C4) variation and those affecting microglial cells in schizophrenia] may increase the risk of excessive microglial activation. Activated microglia produce and release key pro-inflammatory cytokines and free radicals, which have been shown to cause brain structural abnormalities; higher levels of these substances are associated with lower gray matter volumes, especially in brain regions responsible for memory and other cognitive functions. Microglial activation may thus be an important contributor to the development and progression of cognitive impairment in schizophrenia. Its inhibition through the use of microglial inhibitors, such as minocycline and natural products, or the targeting of microglia-expressed receptors, such as with GLP-1R or α7nAChR agonists, holds promise as a potential therapeutic intervention to improve the cognitive function of patients with schizophrenia. GLP-1R, glucagon-like peptide-1 receptor; α7nAChR, α7 nicotinic acetylcholine receptor; IL, interleukin; TNF, tumor necrosis factor.

We propose a potential mechanism of action underlying the relationship between microglial activation and cognitive impairment in patients with schizophrenia (Fig. 1). Stressful psychological and medical (e.g., viral infection, traumatic brain injury) events, which usually precede the onset of psychotic symptoms in patients with schizophrenia and are considered to be important environmental risk factors for the disease, may induce the activation of resting microglia, which release proinflammatory cytokines and free radicals, demonstrated to cause brain structural abnormalities (i.e., GMV loss, especially in memory-processing brain regions)^56–59^. Genetic factors may also increase the risk of microglial activation in schizophrenia^60–66^. The microglia-related genes and genetic variants associated with schizophrenia are summarized in Table 1. Inhibition of microglial activity could be achieved via microglial inhibitors, such as minocycline and other natural products, or the targeting of microglia-expressed receptors, including the glucagon-like peptide-1 receptor (GLP-1R) and α7 nicotinic acetylcholine receptor (α7nAChR) agonists. This approach holds promise as a potential therapeutic intervention to improve cognition in patients with schizophrenia.

**Table 1:** Summary of genes and genetic variants of microglial cells associated with schizophrenia.

Genes	Variants	Putative regulation	References
TNF receptor superfamily member 1 (*TNFR1*)	rs4149577 rs1860545	Up-regulation	^60^^,61,63^
Tumor necrosis factor (*TNF*)	rs1800629	Up-regulation	^61^^,62^^,65^
Interleukin 6 (*IL6*)	rs1800795	Up-regulation	^64^^,66^

### Implications for preventive and therapeutic strategies, and future research directions

Mild to severe cognitive impairment, which affects the majority of patients with schizophrenia, including those taking antipsychotic agents, poses mechanistic and therapeutic challenges^15,18,67,68^. The mechanisms underlying the development and progression of cognitive deficits in patients with schizophrenia are complex. Recent advances in the understanding of the connection between excessive microglial activation, especially in brain regions involved in memory, and schizophrenia-associated cognitive impairment have provided evidence that the suppression of such activation or remodeling of activated microglia may improve cognitive performance in patients with schizophrenia. We also postulate that the inhibition of microglial activation may prevent the development of cognitive decline before the manifestation of psychotic-like symptoms in individuals at high risk of schizophrenia, such as those with first-degree relatives with the disease. Based on the most recent findings that the microglial SZ-PGS predicts variation in measures of cognition across three domains (general cognitive ability, episodic memory, and working memory), and that it correlates inversely with cognitive ability, especially memory function, the most commonly impaired cognitive domain in patients with schizophrenia^14^, the exploration of whether the inhibition of microglial activation in individuals at high risk (according to the SZ-PGS) can prevent the development of cognitive decline at the time of the first psychotic episode, in the prodromal stage, or even before psychotic-like symptom onset would be worthwhile.

Microglial inhibitors, including minocycline and natural products (e.g., apigenin, fisetin, and curcumin), show promise in the treatment of cognitive impairment associated with neuropsychiatric disorders, including schizophrenia^69^. Minocycline, a classical semi-synthetic tetracycline antibiotic with good brain infusion, is used most widely to inhibit microglial overactivation. Clinical studies have demonstrated that the use of minocycline as an add-on drug in combination with antipsychotic medications alleviates negative symptoms (lessening or absence of thoughts and behaviors in relation to interest, motivation, and/or emotional expression) in patients with schizophrenia^70–75^. In an animal model of schizophrenia, minocycline improved the cognitive impairment induced by MK-801, an N-methyl-D-aspartate (NMDA) receptor antagonist^75^. However, concerns have been raised about the detrimental effects of the minocycline treatment, including the promotion of extensive neuronal cell death, disruption of subventricular zone neurogenesis, and induction of synaptic pruning in early postnatal and adolescent rodent brains^74^.

The adverse effects of the inhibition of microglial activation on cognitive impairment might be diminished by the targeting of microglia-expressed receptors (e.g., GLP-1R and α7nAChR agonists), although few studies on this topic have been conducted with patients with schizophrenia or animal models of the disease. Activated microglial cells are of two subtypes with distinct polarization states: the inflammation-induced M1 phenotype and the immunoregulatory M2 phenotype^76,77^. The modulation of microglial polarization has been demonstrated to attenuate neural inflammation–associated pathological events^78^. However, whether microglial phenotype remodeling has a beneficial impact on schizophrenia-related cognitive impairment remains unexplored.

GLP-1, an endogenous enteric peptide, has been shown to play roles in human health and disease through the facilitation of insulin secretion^79^. Various GLP-1R agonists cross the blood–brain barrier, reduce neuronal inflammation, and protect against the cognitive symptoms of Alzheimer’s disease^80,81^. In in-vitro and in-vivo studies, Qian and colleagues^82^ showed that a GLP-1R agonist changed activated M1 microglia to the M2 subtype, and thus is a potential therapeutic agent for the attenuation of cognitive impairment in patients with schizophrenia.

α7nAChR is expressed widely in neurons and microglia in the brain^83^. As the activation of microglia-expressed α7nAChR induces anti-inflammatory effects, this receptor has been proposed as a promising target for the prevention and treatment of cognitive impairment in patients with psychiatric disorders such as schizophrenia^83,84^. In a phase-II clinical trial, Keefe and colleagues^85^ assessed the efficacy of encenicline, a selective α7nAChR agonist, in the treatment of cognitive impairment in patients with schizophrenia. The cognitive performance was evaluated using the Overall Cognition Index as the primary endpoint and the Schizophrenia Cognition Rating Scale total score; MATRICS Consensus Cognitive Battery score; Positive and Negative Syndrome Scale total, subscale, and cognition factor scores; and Schizophrenia Cognition Rating Scale global rating as secondary endpoints^85^. A 12-week encenicline regimen appeared to improve cognition in a clinically meaningful manner relative to placebo^85^. In two phase-III clinical trials, however, encenicline failed to achieve the primary CogState overall cognition index endpoint in patents with schizophrenia^86^. As the C4 genetic variant is associated with a high risk of microglial activation, clinical trials examining the efficacy of encenicline in improving cognitive impairment in patients with schizophrenia and this variant should be conducted.

Sodium benzoate, a metabolite of cinnamon and widely used food preservative, has been shown to reduce the microglial inflammatory response in mice^87^. In randomized, double-blind, placebo-controlled clinical trials, adjunct treatment with sodium benzoate (1 g/day) alone and in combination with sarcosine resulted in improved cognition (processing speed and visual learning), with good tolerance and favorable safety profiles, in patients with schizophrenia^88,89^.

Future directions for microglia-targeting strategies involve the examination of the therapeutic potential of drugs such as minocycline and GLP-1R agonists such as tirzepatide (a dual agonist of the receptors of two insulin-sensitizing polypeptides, GLP-1 and glucose-dependent insulinotrophic polypeptide, recently approved as an adjunct to diet and exercise for the treatment of diabetes, obesity, and non-alcoholic fatty liver disease) in terms of the remodeling of activated microglia from the M1 to the M2 phenotype Future clinical studies should also be conducted with suitable cohorts of patients with schizophrenia, such as those with the C4 variant, associated with a high risk of microglial activation. In view of sex differences in the epidemiology and pathology of schizophrenia, the incidence of which is moderately higher in males than in females, sex-specific genetic factors need to be considered when planning future etiological and clinical studies of cognitive impairment in patients with this disease.

## Conclusions

In conclusion, microglia, a unique lineage of macrophages in the brain, play at least a partial role in cognitive impairment in schizophrenia. Although current knowledge about the role of microglial activation in the onset, development, and worsening of cognitive deficits in patients with schizophrenia remains very limited, we propose a hypothetical mechanism of the action of microglia in the pathogenesis of cognitive impairment in schizophrenia based on existing findings (Fig. 1). Stressful events (i.e., psychological stress), known as important environmental risk factors for schizophrenia, may trigger the activation of resting microglia. These activated cells produce proinflammatory cytokines and free radicals, which may be associated with key brain structural changes, mainly in the GMV, particularly in memory-processing areas of the brain. Thus, microglial activation may be an important factor for cognitive impairment in schizophrenia. Its inhibition through the use of microglial inhibitors or targeting of microglia-expressed receptors could be a novel strategy for the treatment or prevention of cognitive deficits in patients with schizophrenia. In future clinical studies, investigation of the therapeutic effects of the inhibition of microglial activation in more suitable cohorts of patients with schizophrenia or individuals with first-degree relatives with schizophrenia and a high risk of microglial activation, such as those with the complement component C4 variant, would be of interest.
